# Supplementary material for: Ten-year in-hospital mortality trends among Japanese injured patients by age, injury severity, injury mechanism, and injury region: A nationwide observational study
Source: PLoS One. 2022 Aug 22;17(8):e0272573. doi: 10.1371/journal.pone.0272573 (PMC9394834; doi:10.1371/journal.pone.0272573)
Supplement: S1 Table — Some fitted into more than one variable. (DOCX) [file pone.0272573.s002.docx]

Table S1. Number of patients with missing data by study year.

| **Variables** | **2009 n = 11,951** | **2010 n = 14,955** | **2011 n = 18,455** | **2012 n = 22,841** | **2013 n = 29,186** | **2014 n = 30,294** | **2015 n = 29,654** | **2016 n = 23,264** | **2017 n = 25,302** | **2018 n = 25,512** |
| --- | --- | --- | --- | --- | --- | --- | --- | --- | --- | --- |
| Unknown age, n (%) | 10 (0.1) | 16 (0.1) | 31 (0.2) | 38 (0.2) | 28 (0.1) | 31 (0.1) | 27 (0.1) | 16 (0.1) | 29 (0.1) | 30 (0.1) |
| Unknown gender, n (%) | 1 (0.01) | 1 (0.01) | 0 | 8 (0.04) | 6 (0.02) | 9 (0.03) | 6 (0.02) | 13 (0.06) | 11 (0.04) | 3 (0.01) |
| Unknown injury mechanism, n (%) | 89 (0.7) | 117 (0.8) | 165 (0.9) | 185 (0.8) | 201 (0.7) | 186 (0.6) | 238 (0.8) | 160 (0.7) | 142 (0.6) | 176 (0.7) |
| Unknown injury severity score, n (%) | 394 (3.3) | 447 (3.0) | 564 (3.1) | 344 (1.5) | 2096 (7.2) | 2146 (7.1) | 1269 (4.3) | 1346 (5.8) | 1111 (4.4) | 1232 (4.8) |
| Unknown survival outcome, n (%) | 1165 (9.8) | 753 (5.0) | 1152 (6.2) | 1118 (4.9) | 2738 (9.4) | 2573 (8.5) | 1772 (6.0) | 888 (3.8) | 1332 (5.3) | 1444 (5.7) |

Some fitted into more than one variable
