# Supplementary material for: Ten-year in-hospital mortality trends among Japanese injured patients by age, injury severity, injury mechanism, and injury region: A nationwide observational study
Source: PLoS One. 2022 Aug 22;17(8):e0272573. doi: 10.1371/journal.pone.0272573 (PMC9394834; doi:10.1371/journal.pone.0272573)
Supplement: S2 Table — ISS, Injury Severity Score. (DOCX) [file pone.0272573.s003.docx]

Table S2. In-hospital mortality trends among injured patients by age groups and ISS groups

|  |  | **2009** | **2010** | **2011** | **2012** | **2013** | **2014** | **2015** | **2016** | **2017** | **2018** | ***p*-value** |
| --- | --- | --- | --- | --- | --- | --- | --- | --- | --- | --- | --- | --- |
| Age 0–4 | ISS 0−15 | 1 (1.7) | 0 | 3 (3.2) | 1 (0.8) | 0 | 2 (1.2) | 1 (0.6) | 1 (0.8) | 1 (0.7) | 2 (1.3) | 0.686 |
|  | ISS 16−25 | 6 (14.6) | 6 (11.1) | 3 (7.3) | 4 (6.4) | 4 (5.3) | 8 (10.7) | 7 (9.6) | 4 (6.5) | 0 | 7 (10.8) | 0.220 |
|  | ISS ≥26 | 5 (50.0) | 7 (58.3) | 7 (43.8) | 7 (38.9) | 6 (26.1) | 10 (55.6) | 5 (35.7) | 3 (23.1) | 6 (26.1) | 9 (56.3) | 0.339 |
| Age 5–14 | ISS 0−15 | 0 | 3 (0.8) | 1 (0.3) | 1 (0.2) | 3 (0.5) | 2 (0.3) | 0 | 0 | 2 (0.4) | 1 (0.2) | 0.311 |
|  | ISS 16−25 | 7 (6.3) | 9 (6.1) | 4 (2.9) | 13 (7.5) | 5 (2.5) | 3 (1.6) | 4 (2.6) | 3 (1.9) | 5 (2.8) | 4 (3.2) | 0.011 |
|  | ISS ≥26 | 15 (30.0) | 17 (25.0) | 19 (19.4) | 14 (18.9) | 29 (23.4) | 20 (20.2) | 21 (20.8) | 18 (23.1) | 19 (23.5) | 9 (16.7) | 0.403 |
| Age 15–24 | ISS 0−15 | 9 (1.2) | 4 (0.4) | 14 (1.3) | 17 (1.3) | 8 (0.5) | 13 (0.8) | 11 (0.7) | 6 (0.5) | 8 (0.7) | 10 (0.8) | 0.124 |
|  | ISS 16−25 | 37 (12.3) | 45 (10.7) | 33 (7.5) | 32 (6.4) | 43 (6.8) | 39 (6.7) | 36 (6.9) | 35 (8.3) | 25 (5.8) | 31 (7.6) | 0.011 |
|  | ISS ≥26 | 105 (36.8) | 130 (37.8) | 124 (34.2) | 127 (30.2) | 122 (28.2) | 116 (26.6) | 140 (31.4) | 118 (34.9) | 95 (27.1) | 107 (31.4) | 0.013 |
| Age 25–34 | ISS 0−15 | 7 (1.2) | 12 (1.7) | 11 (1.3) | 8 (0.8) | 12 (1.0) | 13 (1.1) | 10 (0.9) | 4 (0.5) | 10 (1.1) | 7 (0.8) | 0.113 |
|  | ISS 16−25 | 27 (7.6) | 43 (12.1) | 28 (7.8) | 50 (14.0) | 41 (11.5) | 35 (9.8) | 39 (10.9) | 36 (10.1) | 31 (8.7) | 27 (7.6) | 0.044 |
|  | ISS ≥26 | 100 (39.5) | 131 (43.1) | 108 (36.4) | 127 (38.5) | 141 (39.1) | 115 (35.9) | 108 (32.5) | 85 (33.9) | 101 (38.9) | 78 (34.5) | 0.034 |
| Age 35–44 | ISS 0−15 | 10 (1.7) | 14 (1.8) | 10 (1.0) | 18 (1.5) | 18 (1.2) | 17 (1.2) | 9 (0.7) | 16 (1.6) | 14 (1.3) | 10 (1.0) | 0.178 |
|  | ISS 16−25 | 32 (10.0) | 42 (10.5) | 69 (15.4) | 62 (11.8) | 53 (8.7) | 44 (6.6) | 55 (8.8) | 43 (9.6) | 38 (7.7) | 26 (5.6) | <0.001 |
|  | ISS ≥26 | 86 (37.1) | 149 (45.3) | 176 (41.6) | 166 (39.6) | 151 (36.0) | 164 (36.8) | 168 (36.4) | 120 (35.2) | 107 (32.4) | 94 (33.5) | <0.001 |
| Age 45–54 | ISS 0−15 | 13 (2.5) | 14 (1.9) | 11 (1.3) | 17 (1.5) | 25 (1.8) | 19 (1.3) | 25 (1.6) | 17 (1.4) | 13 (1.0) | 22 (1.6) | 0.143 |
|  | ISS 16−25 | 50 (14.0) | 48 (11.4) | 55 (10.7) | 67 (11.3) | 73 (9.9) | 81 (10.3) | 76 (10.2) | 43 (7.0) | 55 (7.9) | 47 (6.5) | <0.001 |
|  | ISS ≥26 | 88 (43.4) | 118 (42.9) | 139 (40.2) | 151 (37.7) | 171 (40.1) | 170 (36.0) | 158 (33.6) | 139 (33.3) | 133 (33.6) | 144 (30.4) | <0.001 |
| Age 55–64 | ISS 0−15 | 20 (2.6) | 20 (2.1) | 24 (2.1) | 23 (1.5) | 32 (1.8) | 29 (1.6) | 25 (1.5) | 27 (2.2) | 22 (1.6) | 22 (1.6) | 0.139 |
|  | ISS 16−25 | 76 (14.5) | 86 (13.8) | 105 (12.3) | 127 (12.9) | 108 (10.3) | 102 (9.7) | 90 (9.0) | 83 (10.2) | 70 (7.7) | 67 (8.0) | <0.001 |
|  | ISS ≥26 | 138 (42.7) | 185 (48.3) | 184 (40.4) | 189 (37.8) | 177 (32.7) | 167 (32.0) | 204 (35.6) | 141 (32.7) | 145 (34.3) | 149 (32.5) | <0.001 |
| Age 65–74 | ISS 0−15 | 27 (4.2) | 25 (2.8) | 22 (1.9) | 40 (2.5) | 52 (2.6) | 38 (1.7) | 35 (1.5) | 46 (2.6) | 44 (2.2) | 39 (1.9) | 0.013 |
|  | ISS 16−25 | 100 (18.2) | 106 (15.3) | 134 (14.9) | 175 (15.6) | 170 (13.1) | 169 (11.8) | 171 (11.3) | 140 (11.3) | 146 (10.9) | 136 (9.9) | <0.001 |
|  | ISS ≥26 | 116 (41.3) | 183 (46.5) | 218 (44.7) | 254 (42.1) | 268 (41.2) | 249 (36.1) | 258 (36.1) | 234 (37.5) | 210 (32.7) | 215 (32.4) | <0.001 |
| Age ≥75 | ISS 0−15 | 50 (3.4) | 81 (4.2) | 88 (3.5) | 109 (3.1) | 144 (3.1) | 142 (2.8) | 140 (2.8) | 105 (2.7) | 139 (2.9) | 127 (2.6) | 0.001 |
|  | ISS 16−25 | 140 (23.5) | 191 (24.0) | 240 (22.2) | 308 (22.2) | 334 (19.0) | 333 (16.8) | 365 (17.2) | 319 (17.1) | 328 (16.0) | 327 (15.3) | <0.001 |
|  | ISS ≥26 | 191 (59.7) | 250 (55.4) | 322 (55.5) | 352 (50.7) | 401 (49.0) | 370 (43.2) | 436 (44.9) | 352 (41.9) | 389 (41.7) | 398 (43.0) | <0.001 |

ISS, Injury Severity Score
