# Supplementary material for: Ten-year in-hospital mortality trends among Japanese injured patients by age, injury severity, injury mechanism, and injury region: A nationwide observational study
Source: PLoS One. 2022 Aug 22;17(8):e0272573. doi: 10.1371/journal.pone.0272573 (PMC9394834; doi:10.1371/journal.pone.0272573)
Supplement: S3 Table — ISS, Injury Severity Score. (DOCX) [file pone.0272573.s004.docx]

|  |  | **2009** | **2010** | **2011** | **2012** | **2013** | **2014** | **2015** | **2016** | **2017** | **2018** | ***p*-value** |
| --- | --- | --- | --- | --- | --- | --- | --- | --- | --- | --- | --- | --- |
| Blunt injury | ISS 0−15 | 124 (2.4) | 157 (2.3) | 159 (1.9) | 221 (1.9) | 274 (1.9) | 248 (1.7) | 231 (1.6) | 203 (1.8) | 235 (1.9) | 227 (1.8) | <0.001 |
|  | ISS 16−25 | 453 (15.4) | 544 (14.3) | 635 (13.7) | 801 (14.2) | 797 (12.0) | 790 (11.2) | 815 (11.5) | 684 (11.6) | 674 (10.5) | 661 (10.3) | <0.001 |
|  | ISS ≥26 | 830 (43.1) | 1150 (45.6) | 1276 (42.2) | 1364 (40.0) | 1442 (38.5) | 1364 (35.7) | 1479 (36.6) | 1188 (36.2) | 1193 (35.0) | 1182 (34.8) | <0.001 |
| Penetrating injury | ISS 0−15 | 13 (3.6) | 16 (3.6) | 25 (4.7) | 13 (1.9) | 20 (2.7) | 27 (3.7) | 25 (3.4) | 19 (3.7) | 18 (3.0) | 13 (2.4) | 0.385 |
|  | ISS 16−25 | 22 (23.4) | 32 (24.8) | 36 (31.0) | 37 (26.6) | 34 (22.8) | 24 (14.6) | 28 (22.4) | 22 (18.8) | 24 (22.4) | 11 (11.7) | 0.004 |
|  | ISS ≥26 | 14 (45.2) | 20 (51.3) | 21 (47.7) | 23 (46.9) | 24 (51.1) | 17 (40.5) | 19 (46.3) | 22 (44.9) | 1 (40.0) | 21 (51.2) | 0.750 |

Table S3. In-hospital mortality trends by injury mechanism and ISS groups

ISS, Injury Severity Score.
