# Supplementary material for: Ten-year in-hospital mortality trends among Japanese injured patients by age, injury severity, injury mechanism, and injury region: A nationwide observational study
Source: PLoS One. 2022 Aug 22;17(8):e0272573. doi: 10.1371/journal.pone.0272573 (PMC9394834; doi:10.1371/journal.pone.0272573)
Supplement: S4 Table — AIS, Abbreviated Injury Scale; ISS, Injury Severity Score. (DOCX) [file pone.0272573.s005.docx]

Table S4. In-hospital mortality trends by injury region and ISS groups

|  |  | **2009** | **2010** | **2011** | **2012** | **2013** | **2014** | **2015** | **2016** | **2017** | **2018** | ***p*-value** |
| --- | --- | --- | --- | --- | --- | --- | --- | --- | --- | --- | --- | --- |
| Polytrauma | ISS 0−15 | – | – | – | – | – | – | – | – | – | – | – |
|  | ISS 16−25 | 92 (13.5) | 118 (12.7) | 152 (14.0) | 167 (13.3) | 171 (11.4) | 150 (9.5) | 173 (11.6) | 144 (11.4) | 144 (10.4) | 109 (8.1) | <0.001 |
|  | ISS ≥26 | 676 (43.5) | 957 (46.1) | 1025 (43.1) | 1118 (40.6) | 1139 (38.5) | 1079 (36.0) | 1180 (36.8) | 949 (36.2) | 956 (35.1) | 923 (34.7) | <0.001 |
| Head injury with AIS ≥3 | ISS 0−15 | 38 (5.0) | 48 (4.9) | 47 (4.0) | 76 (5.0) | 78 (4.4) | 54 (2.9) | 60 (3.3) | 40 (2.8) | 53 (3.3) | 59 (3.3) | 0.000 |
|  | ISS 16−25 | 273 (20.9) | 296 (18.5) | 358 (18.1) | 453 (18.6) | 462 (16.6) | 469 (15.6) | 477 (15.5) | 403 (16.3) | 406 (15.0) | 403 (14.7) | <0.001 |
|  | ISS ≥26 | 88 (37.9) | 118 (42.0) | 153 (37.5) | 157 (35.2) | 196 (37.6) | 172 (31.6) | 173 (32.3) | 145 (32.2) | 153 (33.5) | 168 (36.6) | 0.022 |
| Facial injury with AIS ≥3 | ISS 0−15 | 1 (5.6) | 0 | 0 | 0 | 0 | 2 (4.1) | 0 | 1 (2.6) | 0 | 0 | 0.582 |
|  | ISS 16−25 | – | 0 | 0 | 0 | 1 (20.0) | 1 (14.3) | 0 | 0 | 0 | 0 | 0.545 |
|  | ISS ≥26 | – | – | – | – | – | – | – | – | – | – | – |
| Neck injury with AIS ≥3 | ISS 0−15 | 3 (12.5) | 6 (18.2) | 3 (7.9) | 3 (7.1) | 4 (9.1) | 10 (21.3) | 5 (9.8) | 8 (17.8) | 6 (14.3) | 4 (9.8) | 0.780 |
|  | ISS 16−25 | 3 (60.0) | 4 (40.0) | 4 (40.0) | 5 (38.5) | 4 (28.6) | 4 (33.3) | 4 (36.4) | 5 (26.3) | 6 (60.0) | 2 (20.0) | 0.400 |
|  | ISS ≥26 | – | 0 | 1 (33.3) | 4 (80.0) | 1 (100) | 1 (50.0) | 2 (100) | – | 0 | 0 | 0.720 |
| Chest injury with AIS ≥3 | ISS 0−15 | 23 (4.3) | 25 (3.7) | 35 (4.5) | 25 (2.6) | 49 (3.9) | 37 (3.1) | 39 (2.9) | 44 (4.2) | 26 (2.5) | 34 (2.8) | 0.054 |
|  | ISS 16−25 | 55 (10.1) | 99 (14.9) | 90 (11.8) | 141 (14.1) | 122 (10.8) | 121 (9.6) | 118 (9.7) | 89 (8.0) | 78 (6.8) | 104 (8.9) | <0.001 |
|  | ISS ≥26 | 56 (54.4) | 66 (62.3) | 79 (54.9) | 84 (60.4) | 106 (57.9) | 90 (44.6) | 103 (56.9) | 82 (54.3) | 69 (48.9) | 94 (50.0) | 0.040 |
| Abdominal and pelvic injury with AIS ≥3 | ISS 0−15 | 7 (4.1) | 10 (4.6) | 7 (3.0) | 8 (2.8) | 12 (3.5) | 10 (3.3) | 9 (3.2) | 7 (3.2) | 11 (4.6) | 12 (4.3) | 0.718 |
|  | ISS 16−25 | 30 (31.9) | 17 (16.8) | 18 (14.6) | 23 (14.8) | 21 (13.8) | 27 (15.5) | 18 (12.2) | 26 (21.1) | 18 (16.7) | 15 (11.9) | 0.033 |
|  | ISS ≥26 | 4 (57.1) | 7 (50.0) | 4 (36.4) | 3 (42.9) | 5 (33.3) | 5 (83.3) | 6 (75.0) | 6 (75.0) | 3 (75.0) | 0 | 0.840 |
| Spinal injury with AIS ≥3 | ISS 0−15 | 2 (0.8) | 7 (2.1) | 7 (1.6) | 8 (1.5) | 13 (1.9) | 16 (2.0) | 14 (1.8) | 10 (1.6) | 17 (2.4) | 11 (1.5) | 0.562 |
|  | ISS 16−25 | 11 (3.4) | 15 (3.3) | 26 (4.2) | 22 (3.1) | 20 (2.1) | 11 (1.2) | 25 (2.5) | 23 (2.9) | 17 (1.8) | 21 (2.4) | 0.022 |
|  | ISS ≥26 | 10 (25.0) | 16 (29.6) | 21 (23.1) | 17 (21.8) | 17 (18.9) | 25 (21.0) | 29 (23.2) | 26 (32.1) | 19 (19.6) | 16 (16.7) | 0.319 |
| Upper extremity injury with AIS ≥3 | ISS 0−15 | 3 (1.1) | 4 (1.0) | 4 (0.8) | 3 (0.5) | 8 (1.0) | 4 (0.5) | 8 (0.9) | 5 (0.7) | 7 (1.0) | 8 (1.0) | 0.804 |
|  | ISS 16−25 | 0 | 2 (11.8) | 1 (5.9) | 0 | 0 | 0 | 0 | 0 | 0 | 0 | 0.011 |
|  | ISS ≥26 | – | – | – | – | – | – | – | – | – | – | – |
| Lower extremity injury with AIS ≥3 | ISS 0−15 | 39 (2.3) | 52 (2.3) | 41 (1.4) | 63 (1.7) | 85 (1.8) | 88 (1.7) | 78 (1.6) | 45 (1.3) | 83 (1.9) | 65 (1.4) | 0.020 |
|  | ISS 16−25 | 11 (12.5) | 24 (16.8) | 22 (13.1) | 26 (13.9) | 28 (12.1) | 31 (14.2) | 28 (10.5) | 14 (7.5) | 27 (14.4) | 15 (7.8) | 0.025 |
|  | ISS ≥26 | 8 (42.1) | 6 (21.4) | 14 (26.7) | 4 (13.8) | 2 (7.7) | 9 (31.0) | 4 (15.4) | 2 (8.7) | 4 (33.3) | 2 (8.0) | 0.006 |

AIS, Abbreviated Injury Scale; ISS, Injury Severity Score.
